# Probabilistic Clustering of the Human Connectome Identifies Communities and Hubs

**DOI:** 10.1371/journal.pone.0117179

**Published:** 2015-01-30

**Authors:** Max Hinne, Matthias Ekman, Ronald J. Janssen, Tom Heskes, Marcel A. J. van Gerven

**Affiliations:** 1 Radboud University Nijmegen, Donders Institute for Brain, Cognition and Behaviour, Nijmegen, The Netherlands; 2 Radboud University Nijmegen, Institute for Computing and Information Sciences, Nijmegen, The Netherlands; Beijing Normal University, CHINA

## Abstract

A fundamental assumption in neuroscience is that brain function is constrained by its structural properties. This motivates the idea that the brain can be parcellated into functionally coherent regions based on anatomical connectivity patterns that capture how different areas are interconnected. Several studies have successfully implemented this idea in humans using diffusion weighted MRI, allowing parcellation to be conducted in vivo. Two distinct approaches to connectivity-based parcellation can be identified. The first uses the connection profiles of brain regions as a feature vector, and groups brain regions with similar connection profiles together. Alternatively, one may adopt a network perspective that aims to identify clusters of brain regions that show dense within-cluster and sparse between-cluster connectivity. In this paper, we introduce a probabilistic model for connectivity-based parcellation that unifies both approaches. Using the model we are able to obtain a parcellation of the human brain whose clusters may adhere to either interpretation. We find that parts of the connectome consistently cluster as densely connected components, while other parts consistently result in clusters with similar connections. Interestingly, the densely connected components consist predominantly of major cortical areas, while the clusters with similar connection profiles consist of regions that have previously been identified as the ‘rich club’; regions known for their integrative role in connectivity. Furthermore, the probabilistic model allows quantification of the uncertainty in cluster assignments. We show that, while most clusters are clearly delineated, some regions are more difficult to assign. These results indicate that care should be taken when interpreting connectivity-based parcellations obtained using alternative deterministic procedures.

## Introduction

The brain can be described as a vast network of interconnected neurons. The acquisition and subsequent analysis of this network has spawned a discipline known as *connectomics* [1, 2]. Part of connectomics focusses on *structural connectivity*, which concerns the layout of physical tracts consisting of axonal fibers. At a macroscopic scale, structural connectivity is defined in terms of brain regions that consist of neuronal populations which are inter-connected via white matter fiber bundles. As neuronal activity is constrained by neuroanatomy, correctly identifying these structural connections aids in understanding how spatially remote regions of the brain cooperate [3, 4]. Both functional and structural connectivity have been shown to be relevant in clinical applications, for instance by characterizing the connectomes that correspond to neurological and/or psychological disorders [5–7]. Similarly, connectivity studies have become a useful aid in understanding cognition by elucidating which networks are related to particular functions [8–11].

An important application of structural connectivity is the delineation of functionally specialized clusters of brain regions based on their structural connectivity patterns. Importantly, connectivity-based parcellation can be obtained *in vivo*, contrasting it with other methods such as histological analysis of cytoarchitecture [12, 13]. Other non-invasive methods that delineate structural cortical boundaries exist, based on identification of major anatomical landmarks, but these approaches have been shown to be susceptible to large inter-subject variability [14]. Intuitively, a cluster consists of a set of regions that are more similar to other regions in the cluster than to regions outside of it, but this idea can be operationalized in different ways.

Under the first interpretation, which we refer to as *profile-based clustering*, regions may be clustered based on similarity of their connectivity profiles (e.g. [15–19], or see [20] for a literature review). In other words, it assumes that regions in the same cluster connect with the same areas. Several studies have used this approach to parcellate regions of interest such as the frontal pole [21], posteromedial cortex [22], occipital lobes [23], cingulate cortex [24] and thalamus [25–27]. Note that in this interpretation of connectivity-based clusters, regions within a cluster are not necessarily mutually connected. Implicitly, this approach aims to find regions that similarly integrate information from other parts of the brain.

Under the second interpretation, which we refer to as *community-based clustering*, parcellations are taken to consist of densely connected clusters that are only sparsely connected to regions outside the cluster. This approach is typically used for whole-brain parcellation [28–32]. Here, one implicitly assumes that clusters are structurally (and thus, indirectly, functionally) specialized and mostly interact with the other regions in their respective cluster.

Either operationalization of connectivity-based clustering is applicable to the human brain. As a consequence, choosing either perspective may hinder finding meaningful clusters that only adhere to the other definition of a cluster. Furthermore, clustering behavior may not be uniform across the brain, and instead may be a mixture of profile-based and community-based clusters. In this study we introduce a probabilistic model that is able to parcellate structural connectivity and is sufficiently flexible to incorporate the different cluster interpretations. The model reveals community-based clusters in the human connectome, but also a set of nodes that are assigned to small profile-based clusters that function as hubs. These small clusters all contain regions that have been labeled the ‘rich club’ [33]. In addition, we find that the whole-brain parcellations which the model provides, are up to par with algorithms that have previously been used to parcellate connectivity. Moreover, in contrast to parcellations obtained with the other approaches, parcellations estimated by the probabilistic model can be obtained without the need to choose the number of clusters in advance. Finally, because the model is probabilistic, it is able to explicitly represent the uncertainty in the obtained parcellations. These visualizations show that the cluster assignment for particular regions is uncertain, indicating that care should be taken when interpreting connectivity-based parcellations obtained using alternative deterministic procedures.

## Probabilistic model for connectivity-based clustering

**Fig 1:**
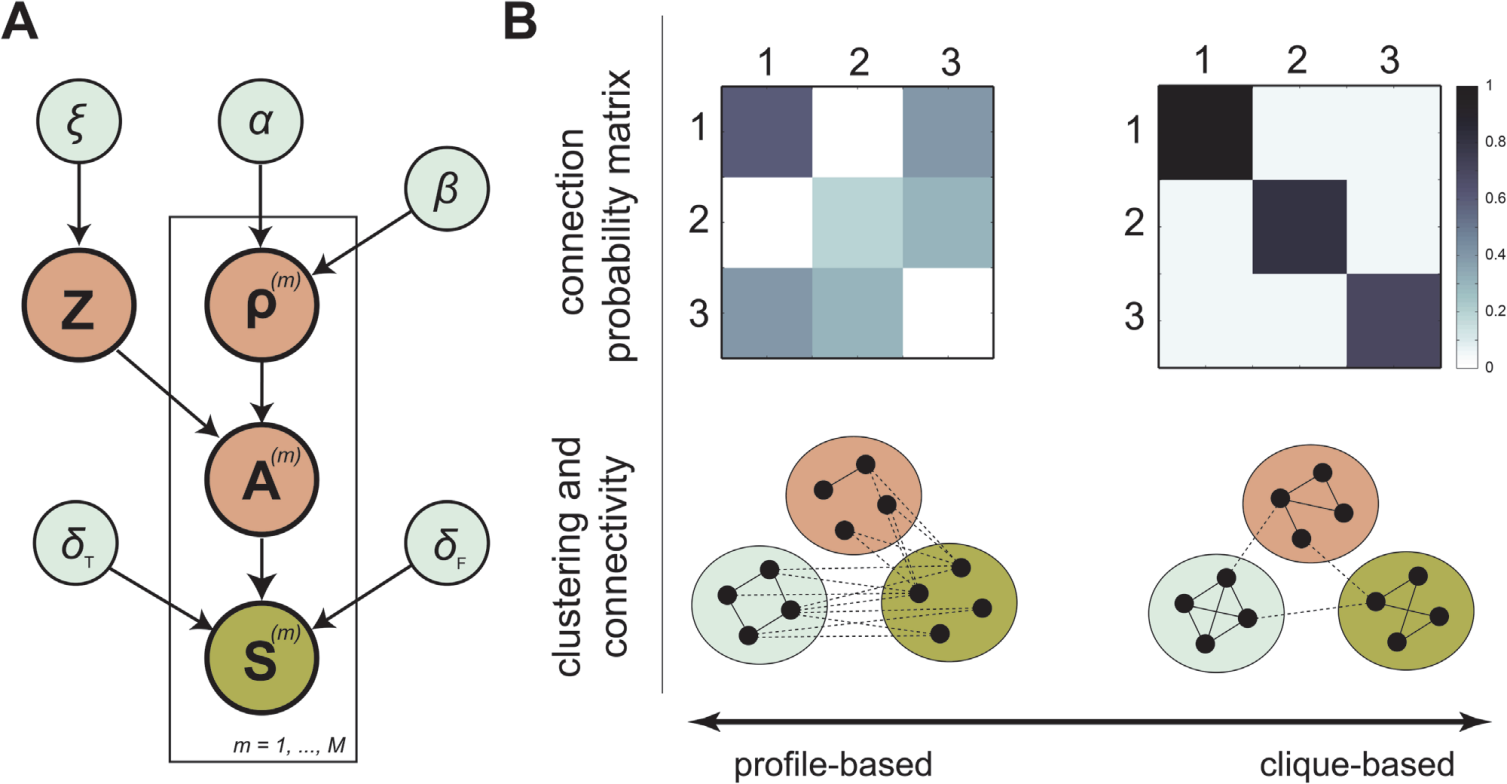
Probabilistic model for connectivity-based parcellation. **A**. The streamline infinite relational model combines a forward model for streamline data **S** with an infinite relational model that allows estimation of the cluster assignment matrix **Z** as well as the connection probability matrix *ρ*. Hyperparameters {*ξ*, *α*, *β*, *δ*_*T*_, *δ*_*F*_} complete the model. **B**. The probabilistic model supports both profile-based clustering as well as community-based clustering. The top row shows simulated connection probability matrices that correspond to profile-based clustering (left) and community-based clustering (right). Example networks that correspond to these probabilities are shown at the bottom.

First, we describe a probabilistic model for clustering based on structural connectivity. Next, we extend this model to allow for direct estimation of cluster structure from probabilistic tractography data. The complete model is shown in Fig. 1A.

### Stochastic block model

Let us assume that each cluster has a distinct connectivity profile. In other words, each cluster has a set of parameters that govern its connectivity behavior. This is known as a stochastic block model (SBM) [34]. Within a cluster, all nodes (i.e. brain regions) use the same connectivity parameters. As a consequence, regions within a cluster are stochastically exchangeable. Formally, each node 1, …, *N* is assigned a cluster label, using the unobserved (latent) cluster assignment variable *Z*_*nk*_ = 1 if node *n* is in cluster *k*, and 0 otherwise. Each node is assigned to exactly one cluster. Structural connectivity is described by a symmetric and binary adjacency matrix **A**. Because of the stochastic exchangeability assumption, the probability of a connection *a*_*ij*_ depends solely on the clusters to which nodes *i* and *j* are assigned. The cluster connection probabilities between clusters *a* and *b* are given by *ρ*_*ab*_. The set of cluster connection probabilities is collected in the connection probability matrix *ρ*. In practice, *ρ* is unknown so we use a prior on *ρ* to reflect our assumptions about it. Here, this is a Beta prior that depends on two hyperparameters *α* and *β*, which model the probability of a connection or non-connection between different clusters, respectively. We assume an uninformative prior with *α* = *β* = 1. After observing the data, the posterior expectation for *ρ* will reflect cluster connection probabilities and therefore informs about the contributions of profile-based and community-based clusters [35]. As an example of how block models may represent different connectivity patterns, Fig. 1B shows the posterior expectations for the connection probability matrix using a toy network of twelve nodes distributed evenly over three clusters. Here, the network on the left shows how profile-based clusters may be captured by the off-diagonal weights of *ρ*, while the network on the right shows how strong weighs on the diagonal of *ρ* generate a traditional community-based network.

SBM have seen widespread application in literature, ranging from discovery of roles in social networks [34] to identification of protein-protein interactions [36, 37]. In addition, several model variants have been introduced, such as approaches that deal with overlapping clusters [38] or SBM tailored to weighted networks [39]. However, these approaches assume that the number of clusters *K* is known a priori, which is frequently not the case with empirical data. A nonparametric extension was introduced to learn the number of clusters from data as well [40]. This is achieved by placing a prior distribution on the cluster assignment matrix **Z**. Doing so allows the model to accommodate a (potentially) infinitely large number of clusters, rather than needing to specify the number of clusters *K* beforehand. Specifically, we draw **Z** from a Chinese restaurant process (CRP) [41]. This distribution over partitions can be used to generate samples from in the following way. Consider nodes that are assigned to clusters one by one, as customers entering a restaurant and choosing a table to sit at. Each customer is assigned to a non-empty table *k* with probability $\frac{m_{k}}{N-1+\xi }$, with *m*_*k*_ the number of customers already assigned to table *k* and with probability $\frac{\xi }{N-1+\xi }$ to an empty (new) table. Its concentration parameter *ξ* determines how likely it is for a customer to sit at an empty table, which affects the total number of tables with customers, i.e. the number of clusters. Using the CRP, the generative model is then given by

$$\begin{array}{ccc} Z|\xi & ∼ & \text{CRP}(\xi )\\ \rho_{ab}|\alpha ,\beta & ∼ & \text{Beta}(\alpha ,\beta )\\ a_{ij}|\rho_{ab},Z & ∼ & \text{Bernoulli}(z_{i}\rho z_{j}^{T})\;, \end{array}$$ (1)

where we introduce the notation **m**_*i*_ to indicate the *i*th row of a matrix **M**. The model is known as the infinite relational model (IRM) [35, 40, 42, 43]. The infinite relational model is easily generalized to encompass multiple (conditionally independent) subjects that share a parcellation [42] by changing the model into

$$\begin{array}{ccc} \rho_{ab}^{\left(m\right)}|\alpha ,\beta & ∼ & \text{Beta}(\alpha ,\beta )\\ a_{ij}^{\left(m\right)}|\rho_{ab}^{\left(m\right)},Z & ∼ & \text{Bernoulli}(z_{i}\rho^{\left(m\right)}z_{j}^{T}) \end{array}$$ (2)

where the superscript *m* indicates the subject index. Note that although the clustering **Z** is shared across subjects, the cluster-to-cluster connection probabilities are subject-specific. When the IRM is applied to binary connectivity data, we will refer to the model as the bIRM.

### Forward model for streamlines

To infer cluster assignments, structural connectivity data must be provided in the form of a binary adjacency matrix **A**. This matrix can be obtained using probabilistic tractography [44]. Specifically, probabilistic tractography proceeds by drawing streamlines between brain regions based on local estimates of anisotropic diffusion. These streamlines are collected in a streamline count matrix **S**, reflecting the number of streamlines between pairs of brain regions. Thresholding of the streamline count matrix produces a binary matrix that reflects structural connectivity. However, as a threshold results in a point estimate, we make use of a probabilistic model that describes how a structural network generates the distributions of probabilistic streamlines that are obtained through tractography [45]. Using this forward model, a streamline threshold is no longer required.

Ideally, probabilistic streamlines show a distribution that perfectly reflects the underlying structural connectivity. However, tractography is prone to noise and errors, in particular in the presence of kissing, splaying and crossing fibres [46]. Hence, we distinguish between true connections along which we expect to observe streamlines, and false connections that may occasionally display streamlines, but do not correspond to actual anatomical pathways. The probability of a streamline between a pair of regions is represented by the matrix **X**. Formally, the streamline probability vector **x**_*i*_ for a particular region *i* is determined by a Dirichlet distribution with parameter *δ*_*T*_ for true connections and *δ*_*F*_ for false connections. According to these probabilities, streamlines are distributed amongst the target regions using a multinomial distribution. By integrating out the streamline probability vectors, we obtain the following forward model:

$$\begin{array}{ccc} s_{i}|a_{i},\delta_{T},\delta_{F} & ∼ & \text{DirMul}(\delta_{T}a_{i}+\delta_{F}\left(1_{n}-a_{i}\right)) \end{array}$$ (3)

where DirMul(*α*) stands for the Dirichlet compound multinomial distribution with hyper-parameters *α*. Note that this formulation assumes undirected structural connectivity, but allows streamline counts *s*_*ij*_ and *s*_*ji*_ to be different.

To estimate connectivity from streamline data, (3) is supplied with a prior on **A**. In the most straightforward case, this prior is uniform, i.e. *P*(**A**) ∝ 1 [45]. Alternatively, this uninformative prior may be replaced by the IRM (see Fig. 1A), expressing our assumption of clustering in the network. The interpretation of the integrated model is that most observed streamlines indicate a structural connection (although there is some noise in the tractography process), and regions in the same cluster share their connectivity preferences (although some exceptions are allowed). We refer to the combined model that operates on streamline data as sIRM. Details of the approximate inference algorithm used to compute posterior estimates of interest are provided in SI1.

### Visualizing clustering uncertainty

In addition to the display of individual parcellations, such as the maximum a posteriori (MAP) estimate, the proposed method may be used to visualize uncertainty about a parcellation. However, as the number of clusters may vary within a clustering distribution, and cluster labels are arbitrary for each sample, such a visualization is not trivial. A representation that allows different samples to be compared is the cluster co-assignment matrix **M** = **Z** ^*T*^ **Z**. The expectation of **M**, i.e. $E[M]=\frac{1}{S}\sum_{s=1}^{S}M_{s}$, with *S* the number of obtained samples (see SI1), describes the posterior probability that two regions are assigned to the same cluster. The probability of co-assignment is used to color a region *i* with a weighted color coding given by $c_{i}=\sum_{j}m_{ij}{\hat{c}}_{j}/\sum_{j}m_{ij}$, with ${\hat{c}}_{j}$ the color representation in the MAP estimate for region *j*. For example, suppose that the MAP estimate consists of two clusters, one colored red and one colored yellow. If a region *i* is co-assigned to a region in the red cluster in half the samples, and to a region in the yellow cluster in the other half of the samples, it will be colored orange.

### Evaluating parcellation quality

Since a clustering ground truth is unavailable, we used the reproducibility of the parcellations as a indicator of parcellation quality [14, 47, 48]. We quantify the reproducibility as the similarity between the parcellations of different participants (or groups of participants), expressed using adjusted mutual information (AMI) [49]. The AMI measure differs from the more traditional normalized mutual information measure in that it compensates for possible bias as a result of different numbers of clusters per parcellation. The measure is defined as

$$S\left(Z_{1},Z_{2}\right)=\frac{\mathrm{MI}\left(Z_{1},Z_{2}\right)-E\left[\mathrm{MI}(Z_{1},Z_{2})\right]}{\max\left(H\left(Z_{1}\right),H\left(Z_{2}\right)\right)-E\left[\mathrm{MI}(Z_{1},Z_{2})\right]}\;,$$ (4)

with MI(**Z**_1_, **Z**_2_) the mutual information between two clusterings, H(**Z**) the entropy of a clustering and $E\left[\text{MI}(Z_{1},Z_{2})\right]$ the expected mutual information between two clusterings.

For comparison, we also obtain parcellations using *K*-means, the canonical algorithm for profile-based clustering, and the Infomap algorithm [50], which relies on community-based clustering (see SI3). Both methods have been used before in the context of brain parcellation [18, 32]. Each of the comparison algorithms was applied to the empirical streamline distributions. For *K*-means and Infomap the streamline matrices were made symmetric, i.e. **S** ^′^ = **S** + **S** ^*T*^. The number of clusters for *K*-means and Infomap is fixed to be the same as for the MAP estimate of the sIRM approach. To apply the bIRM, we first obtained the MAP estimate of connectivity with a flat prior, i.e. *P*(**A**) ∝ 1, which was subsequently clustered using the IRM approach. For the group-level analysis, a group-level streamline matrix was created by summing the streamline counts of all ten participants per split. This matrix was provided as input for *K*-means and Infomap. The bIRM method uses the ten individual MAP estimates of the forward model as input, while the sIRM method uses the ten streamline matrices.

## Results

The sIRM was used to obtain posterior distributions as well as MAP estimates for twenty participants, using the parameter settings described in SI2. On average, the MAP clusterings of these participants consisted of 12.10 (SD 1.29) clusters. The posterior distributions of the numbers of clusters are centered tightly around the MAP estimates, as evidenced by a mean range of the 95% credible intervals of only 0.85 (SD 0.81) (i.e. most of the samples of the approximated distribution have the exact same number of clusters). To analyze the behavior of group-level parcellations, we created 20 random splits of the set of subjects and obtained the MAP parcellation for all of the 40 halves. These 40 parcellations consisted on average of 15.03 (SD 0.83) clusters. At both the individual and the group-level we find that all identified clusters are spatially contiguous, with the exception that the superior frontal gyrus and the precuneus are sometimes assigned to the same cluster as their functional homologue in the contralateral hemisphere.

**Fig 2:**
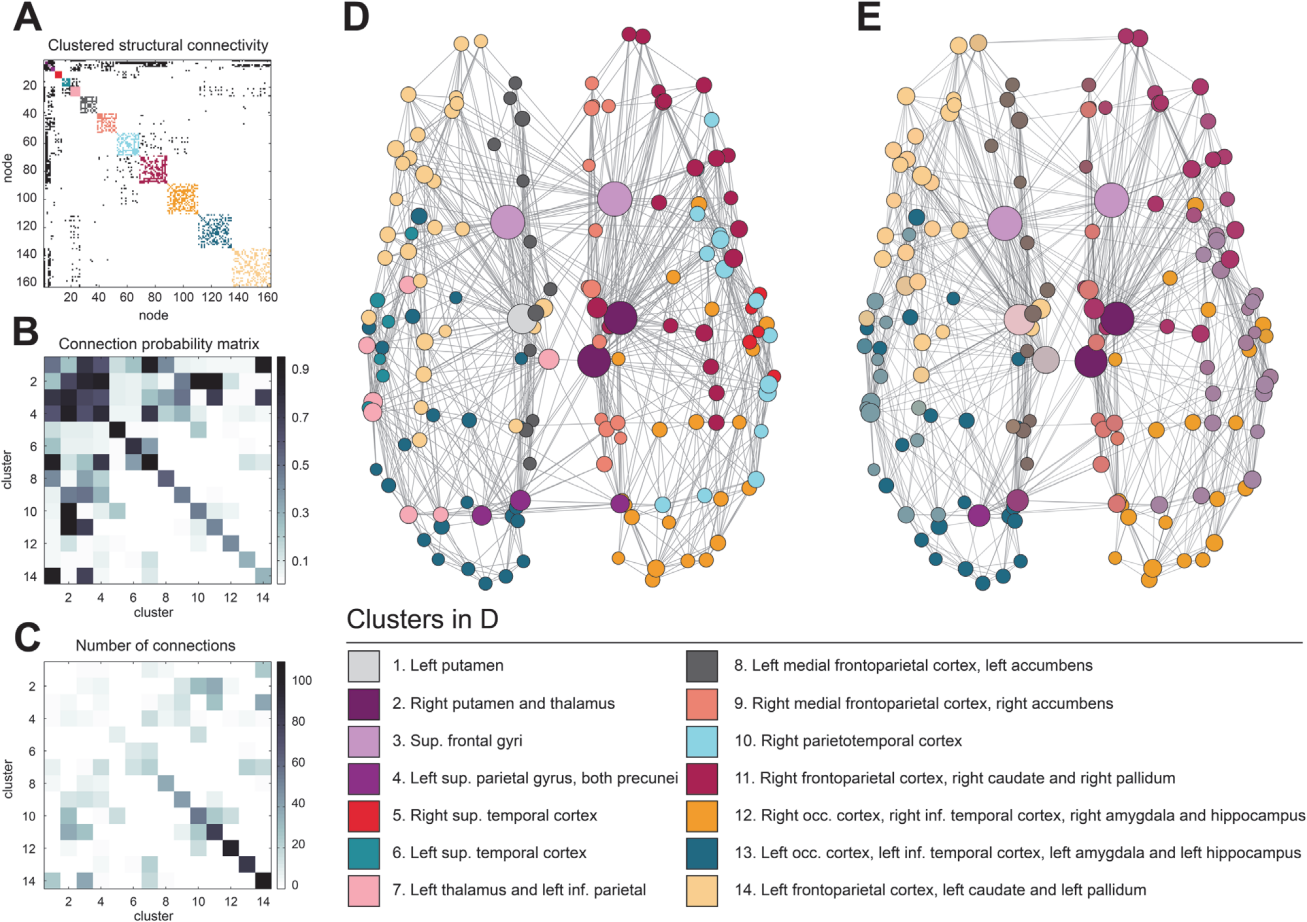
(Color online) Maximum a posteriori parcellations for one participant. **A.** The adjacency matrix **A**. **B.** The connection probability matrix *ρ*. **C.** The number of connections between clusters **ZAZ** ^*T*^. **D.** Visualization of the maximum a posteriori network structure and parcellation. The clusters are color coded to be able to compare the network with the adjacency matrix in **A.** Node sizes are scaled by their degree. **E.** Visualization of the expectation of network structure and parcellations. Colors are interpolated with the MAP estimate as point of reference (see text). To keep the visualization uncluttered, only the *m* most probable edges are shown, where *m* is the number of edges in the MAP estimate.

**Fig 3:**
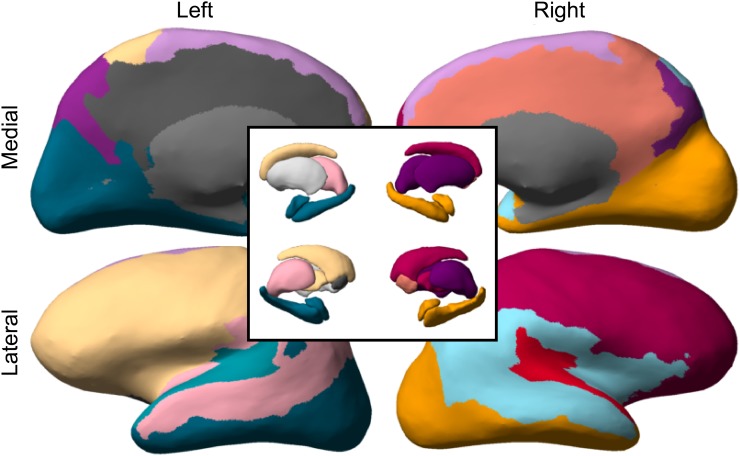
(Color online) The maximum a posteriori parcellation for one participant (see Fig. 2), projected onto the inated cortical surface and subcortical areas. The coloring corresponds to the colors used in Fig. 2.

As an example, the MAP estimate for one participant is shown in Fig. 2A–D and Fig. 3. The other MAP estimates are shown in S2 Fig. and S3 Fig. for the individual participants and the 40 halves, respectively. Fig. 2A shows the connectivity matrix **A**. Within-cluster connections are colored with the color of their respective clusters and between-cluster connections are colored black. Fig. 2B shows the probability of a connection between pairs of clusters, as represented by the connection probability matrix *ρ*. The number of connections between pairs of clusters, **ZAZ** ^*T*^, is shown in Fig. 2C. Note that the number of possible connections grows with $m_{k}^{2}$, so while the amount of connection increases, the cluster connection probability may decrease. A visualization of the layout of the connectivity **A** and clustering **Z** of this MAP estimate is shown in Fig. 2D.

**Fig 4:**
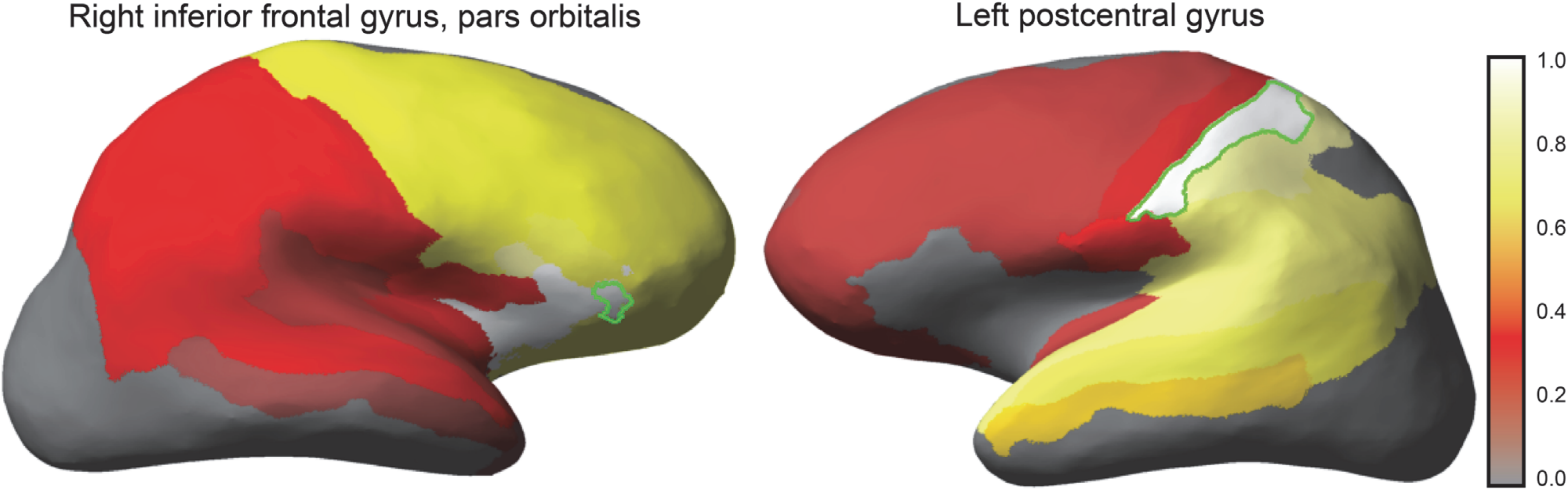
Cluster probability map to visualize the uncertainty of the resulting cluster assignments. (left) Cluster probability for the inferior frontal gyrus. The map shows that the highlighted region is likely to be assigned to regions in the frontal cortex, and to a much lesser degree to regions in the parietal cortex. The opposite pattern is shown for the postcentral gyrus (right).

Fig. 2E shows the interpolated clustering colors based on the approximated posterior distribution of **Z** for this participant. The figure reveals that although the expectation is not very different from the MAP estimate, there remains some room for uncertainty. For instance, the right parietotemporal cortex and the right superior temporal cortex are seen as separate clusters in the MAP estimate, but appear to be merged into a single cluster in a substantial part of the distribution. Further uncertainty is shown in the assignment of left thalamus, right precuneus and left inferior parietal cortex. Zooming in on these regions results in what we will refer to as a ‘cluster probability map’; a map that, for any given region of interest *i*, shows the probability that it is assigned to the same cluster as another region. This corresponds to a row **m**_*i*_ of **M**. Fig. 4 visualizes the cluster probability maps for the right inferior frontal gyrus and the left postcentral gyrus, for the same participant as shown in Fig. 2. Maps like these serve as further illustration that there may be substantial uncertainty in cluster assignments, and that point estimates should be used with care.

### Comparison with other methods

The parcellations for *K*-means, Infomap and bIRM are shown in S4 Fig.–S6, respectively, together with the connections that correspond to the top 5% streamline counts. For the bIRM approach, this resulted in 8.25 (SD 0.55) clusters. At the group level, 10.18 (SD 0.45) clusters were found for the bIRM.

**Fig 5:**
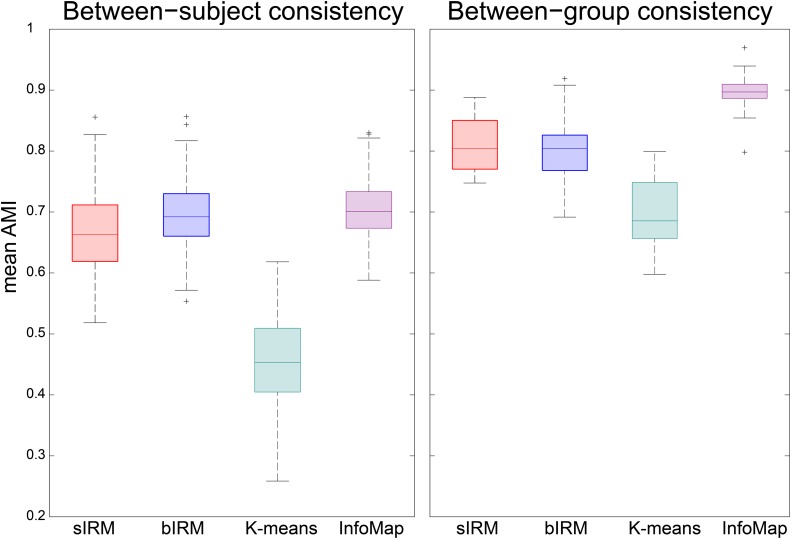
The reproducibility of parcellations. **A.** Mean adjusted mutual information (AMI, see main text) for pairwise comparisons between parcellations of all participants. **B.** Mean AMI for each of the pairs of parcellations that were created by randomly splitting the participant group into halves and obtaining a parcellation for each half.

The results of the reproducibility comparisons are shown in Fig. 5. At the level of individual parcellations, only *K*-means performs notably less consistent than the other methods. The sIRM results in sparse connectivity (the MAP estimates of the twenty subjects have a density of ±7%, on average) and its parcellations are up to par with the bIRM and Infomap. At the group level, Infomap appears to be the most consistent method and *K*-means is again the method that is least consistent. The group-level parcellations obtained by the sIRM are similar in consistency to those obtained by the bIRM.

### Community-based versus profile-based clusters

The MAP estimates of *ρ* as well as the number of connections between clusters in 2 show that clustering behavior depends strongly on cluster identity and that some clusters show community-like tendencies while others do not. To quantify the extent to which a cluster forms a community, and to be able to compare this with the other methods, we computed for each cluster *c* the ratio *r*_*c*_ of within-cluster streamlines versus the total number of streamlines connected to this cluster. When this ratio approaches 1, the corresponding cluster is a community. If instead the ratio approaches 0, it can be regarded a profile-based cluster. As the number of clusters can be different between the sIRM and the bIRM methods, we then assigned to each node the score *r*_*c*_ its corresponding cluster had.

**Fig 6:**
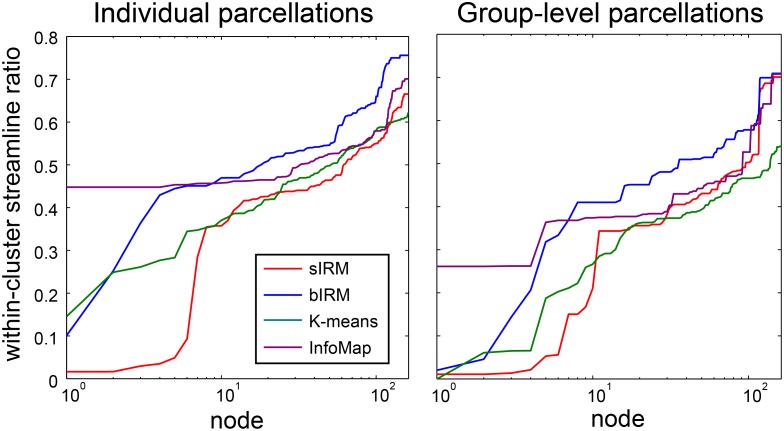
The ratio describing how community-like the clusters are that each region is assigned to. The plots show, for the cluster each region is assigned to, the ratio of within-cluster streamlines versus the total number of streamlines connected to that cluster, for parcellations of individual participants (left) and group-parcellations (right). This reveals that in particular for the sIRM method, this ratio is small for a number of regions, indicating that these regions are not part of community-based clusters. Regions that consistently have a low ratio, are described in the text. Note that the nodes corresponding to each line are ordered differently.

Fig. 6 shows for both the single-participant as well as for the group-level parcellations the ratio *r*_*c*_ for all nodes, as averaged over all participants and all group-level parcellations, respectively. The results for the sIRM method clearly reveal that parcellation can be divided into two regimes. The first consists of clusters with relatively high *r*_*c*_ values (up to 0.76). These are large clusters that correspond predominantly to major cortical areas or lobes that are highly intraconnected. They are connected via the second regime with low *r*_*c*_ values (as low as 0.02), that contains small clusters (containing less than five nodes) and have few within-cluster streamlines. In at least 15 out of 20 participants, the small clusters contain the bilateral putamina, bilateral superior frontal gyri and right thalamus. For the group-level parcellations we observe similar behavior. Most clusters tend to be communities, connected via small clusters consisting of one or two regions. Small-cluster regions that occur in at least 26 out of 40 group-level parcellations are the bilateral precunei, superior frontal gyri, thalami, putamina and left superior parietal gyrus.

The other approaches show different patterns. For the single-participant parcellations, bIRM, *K*-means or Infomap show no regions that consistently appear in clusters with low *r*_*c*_. For group-level parcellations, bIRM finds bilateral superior frontal gyri and right putamen (a subset of the regions found by the sIRM), *K*-means finds right occipital gyrus, right orbital sulcus and right medial olfactory sulcus and Infomap finds bilateral pallida and left putamen. Remarkably, the regions that stand apart according to the sIRM approach (for both the single-subject and the group-level parcellations) are all known to be part of the ‘rich club’ regions that integrate more remote cortical regions [33]. Note that these regions do not mutually form a single large cluster, as each of them has substantially different connections to the rest of the brain.

## Discussion

Human brain connectivity is shown to exhibit clustering according to two different principles. Multiple brain regions show community-based clustering where clusters are both spatially contiguous and densely intraconnected. These clusters are tied together by brain regions that reveal connectivity-based clustering. The latter contain only one or two nodes and do not ‘fit in’ with other clusters due to substantially different connection profiles. These clusters consist predominantly of the superior frontal gyri, the superior parietal gyri, the precunei, the thalami and the putamina. All these regions have previously been identified as members of the ‘rich club’ [33]. In addition, all regions except for the thalamus have been pointed out as being the most vulnerable and central regions in structural brain networks [51]. Since the thalamus can be considered to act as a relay station, it stands to reason that it connects different cortical clusters. The rich-club regions stand apart from the rest of the connectome, as they are not part of the community-like clusters. Rather, they are each assigned their own cluster or group together with a similar region. Intuitively, this is an appealing observation. The community-like clusters contain regions dedicated to specialized processing. Here, an abundance of local connectivity, required for extensive within-cluster communication, results in dense intra-cluster connectivity. Simultaneously, the signal from these clusters must be integrated and disseminated, which is presumably achieved via the rich club regions [52]. Note that although small clusters consisting of one or a few regions will not be community-based clusters by definition, because there are simply not enough possible internal edges, it is the finding that these regions are not assigned to bigger clusters containing other nodes that is interesting. This means that a substantial fraction of the connections of these regions is to several different clusters, which emphasizes their integrative role.

Validating the empirical results from connectivity-based parcellation remains a difficult task [20]. Since ground truth is not available, reproducibility across subjects is often used as a proxy for parcellation quality [14, 47, 48]. Based on this criterion, we have shown that our approach performs well. Yet, by visual inspection of the parcellations (see S2 Fig., S4–S6) some pairs of regions appear grouped together that are not immediately obvious. For instance, in many parcellations we find that thalamus and putamen are assigned to the same cluster, as well as amygdala and hippocampus and bilateral superior frontal gyri. One may argue that in particular for the subcortical areas, these regions should appear in separate clusters as they have specialized functionality. However, since these parcellations are based on connectivity and these regions project similarly to the cortex, they are put together in a cluster. This is inherent to connectivity-based clustering and occurs for *K*-means and Infomap as well. In fact, of the algorithms we considered, sIRM appears to be the only method that consistently assigns many of the rich club regions to small clusters instead of agglomerating them into large clusters. Finally, we note that anatomical constraints such as enforcing that subcortical areas constitute singleton clusters may easily be added into the prior distribution.

Previously used methods are limited to one particular clustering behavior. A notable exception is a recent study that reveals that the connectome of the C. elegans roundworm also consists of a number of densely intraconnected clusters that are integrated via a core cluster that strongly connects to each community [53]. As we described above, the human connectome reveals both densely connected clusters as well as disconnected clusters with very similar connectivity. Consequently, picking one perspective inevitably neglects part of the available structure, which is exemplified by the parcellations found using *K*-means and Infomap that assign the rich club regions to larger clusters. Although, in the case of Infomap, this results in more consistent parcellations, the more detailed picture of communities and integrating regions is lost.

Estimating parcellation with the sIRM approach provides a number of additional interesting quantities apart from the parcellation itself. The cluster connection probabilities *ρ* act as a cluster-level estimation of connectivity, expressed in terms of the rich-club regions and major cortices. Region-to-region connectivity **A** is estimated alongside clustering, and is potentially less prone to noise due to the prior that encourages regions in the same cluster to connect similarly, unless the data provides strong evidence to the contrary. Furthermore, other connectivity-based parcellation strategies, such as *K*-means, which groups regions with similar connectivity, and Infomap, which optimizes for densely connected components, provide point estimates of a parcellation. Although these approaches have provided valuable insight in the organization of both structural and functional connectivity [32, 54, 55], they do not quantify the uncertainty in their results (this issue is also discussed in [48]). Instead, our proposed method characterizes the full posterior distribution of all variables involved and thus provides a richer representation of parcellation. We find that a number of regions show substantial uncertainty in their assignments to a cluster. This illustrates that point estimates should be used with care, as a number of regions could easily be assigned erroneously.

Integrating the forward model for structural connectivity with the IRM as prior leads to qualitatively different parcellations than when the bIRM is applied post-hoc to the connectivity estimates from the forward model. This is visible from the different number of clusters that both pipelines provide (the combined approach results in roughly 50% more clusters). In addition, the clear distinction between the two kinds of clusters is only marginally visible in the bIRM parcellations. Note that regardless of these differences, the reproducibility of the parcellations is similar for both methods.

The IRM is a nonparametric method, which is helpful since the number of clusters is not known a priori. Still, parameters remain that affect the resulting parcellation. In particular the parameters *δ*_*T*_ and *δ*_*F*_ that govern the estimated connectivity and concentration parameter *ξ* that affects the number of identified clusters (although we observe that its influence is drowned out by the contribution of the likelihood) may be estimated from the data using empirical Bayes [56]. However, this will incur substantial additional computation costs that must be overcome to arrive at an efficient model.

There are a number of directions one could take the proposed approach. To start with, alternative generative models may be used instead. For instance, the forward model that now consists of a Dirichlet compound multinomial distribution, modeling the probabilities of streamlines, may be replaced by a Poisson model [57], modeling streamline counts instead. Additional analyses will be needed to identify which model best captures the underlying connectivity. Furthermore, alternative stochastic block models may be used, such as described in a recent study [35] that applies the IRM and two variants to resting-state functional MRI.

Lastly, an interesting avenue to pursue is identifying what causes the uncertainty in the posterior distribution of parcellation. Presumably, this can be attributed to a large extent to noise in data acquisition and tractography. But aside from methodological reasons, there may be other causes that could provide insight in the functional organization of the brain. For instance, uncertainty may in some cases be a result of overlapping clusters [58, 59]. It is likely that regions that are part of multiple, overlapping clusters show higher uncertainty in their cluster assignments. This may be clarified by embedding the infinite relational model within a larger framework to infer clusters at different levels of a nested hierarchy [30, 60]. One would expect that those clusters that show overlap and uncertainty become merged at a higher level of the hierarchy.

## Conclusion

In this paper, we have described an approach for connectivity-based parcellation that encompasses both community-like clusters as well as clusters that have similar connections, without being densely intraconnected. We find that both kinds of clusters are represented in the human connectome, and that the division into these two types corresponds to previous knowledge of structural connectivity. The model is able to quantify which regions are difficult to assign to a cluster, and it learns the number of clusters from the data. Finally, it does not depend on thresholded connectivity, but derives connectivity simultaneously with the parcellation. We hope that connectivity-based parcellation based on probabilistic models such as the one presented here will help to better understand the structural organization principles of human brain networks.

## Materials and Methods

### Ethics statement

Twenty healthy volunteers were scanned after giving informed written consent in accordance with the guidelines of the local reviewing committee CMO Arnhem-Nijmegen. This study was approved by CMO Arnhem-Nijmegen (CMO 2001/095 and amendment “Imaging Human Cognition”).

### Data acquisition

The data used here was previously described in [61]. A T1 structural scan and diffusion-weighted images were obtained using a Siemens Magnetom Trio 3T system at the Donders Centre for Cognitive Neuroimaging, Radboud University Nijmegen, The Netherlands. An optimized acquisition order described by [62] was used in the DWI protocol (voxel size 2.0 mm isotropic, matrix size 110×110, TR = 13000 ms, TE = 101 ms, 70 slices, 256 directions at b = 1500 s/mm^2^ and 24 images at b = 0).

### Preprocessing

DWI data were preprocessed using FSL FDT [63], which consisted of motion correction, correction for eddy currents and estimation of the diffusion parameters. To obtain a measure of white-matter connectivity, we used FDT Probtrackx 2.0 [63, 64] using seed voxel to target voxel tracking. Structural scans were segmented using FAST [65] and FIRST [66] to generate seed and target voxels. Seed voxels were those voxels in the cortical gray matter mask with a non-zero white-matter partial volume estimate and the outermost voxels of the subcortical masks. The remainder of the cortical and subcortical voxels served as target voxels. All FSL preprocessing was performed using version 5.0 and default settings unless otherwise specified. In addition, streamlines were terminated once they hit the target mask. This prevents transitive connections being erroneously interpreted as direct connections.

The cortical surface was reconstructed using Freesurfer version 5.0 [67] and network nodes were defined according to the atlas provided in [68] (see S1 Fig.) in combination with subcortical segmentation using FIRST [66]. This resulted in a total of 162 nodes. Seed and target voxels were assigned to the nearest vertex in the reconstructed surface and by extension to the corresponding region. All streamline counts between two regions were summed to construct the final streamline matrix.

## Supporting Information

S1 Fig The Freesurfer template that defines the network nodes. (EPS)

S2 Fig The participant-level parcellations for the streamline IRM. (TIF)

S3 Fig The group-level parcellations for the streamline IRM. (TIF)

S4 Fig The participant-level parcellations for the *K*-means algorithm. (TIF)

S5 Fig The participant-level parcellations for the Infomap algorithm. (TIF)

S6 Fig The participant-level parcellations for the IRM using the MAP estimates of the connectivity forward model as input. (TIF)

S1 Text Approximate inference of the model. Description of the block Gibbs sampler used to approximate the posterior distributions and maximum a posteriori estimates.
(PDF)

S2 Text Parameter selection. (PDF)

S3 Text Details regarding *K*-means and Infomap. (PDF)
