# Supplementary figures and images for: Probabilistic Clustering of the Human Connectome Identifies Communities and Hubs
Source: PLoS One. 2015 Jan 30;10(1):e0117179. doi: 10.1371/journal.pone.0117179 (PMC4311978; doi:10.1371/journal.pone.0117179)

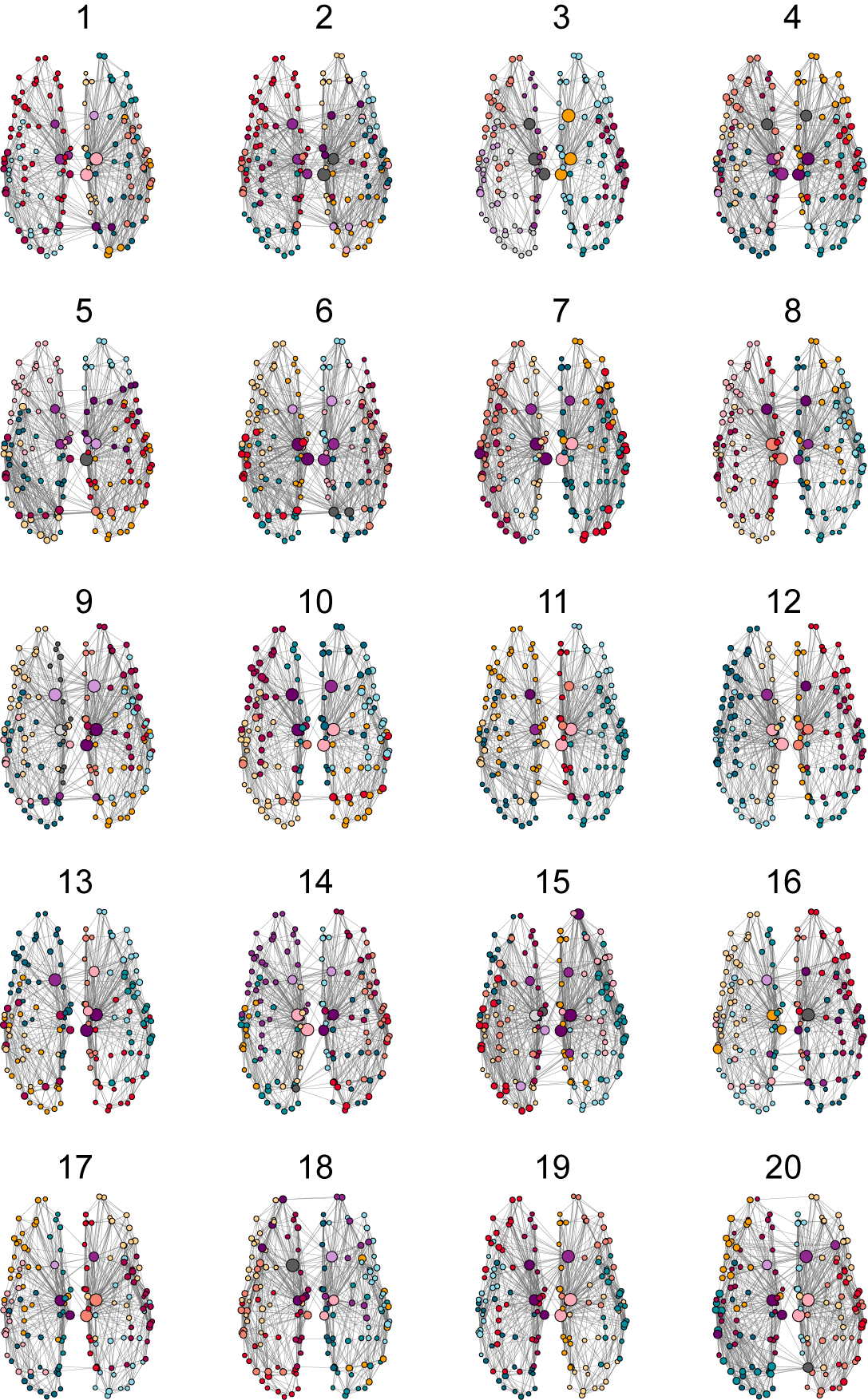

Supplement: S2 Fig — (TIF) [file pone.0117179.s002.tif]

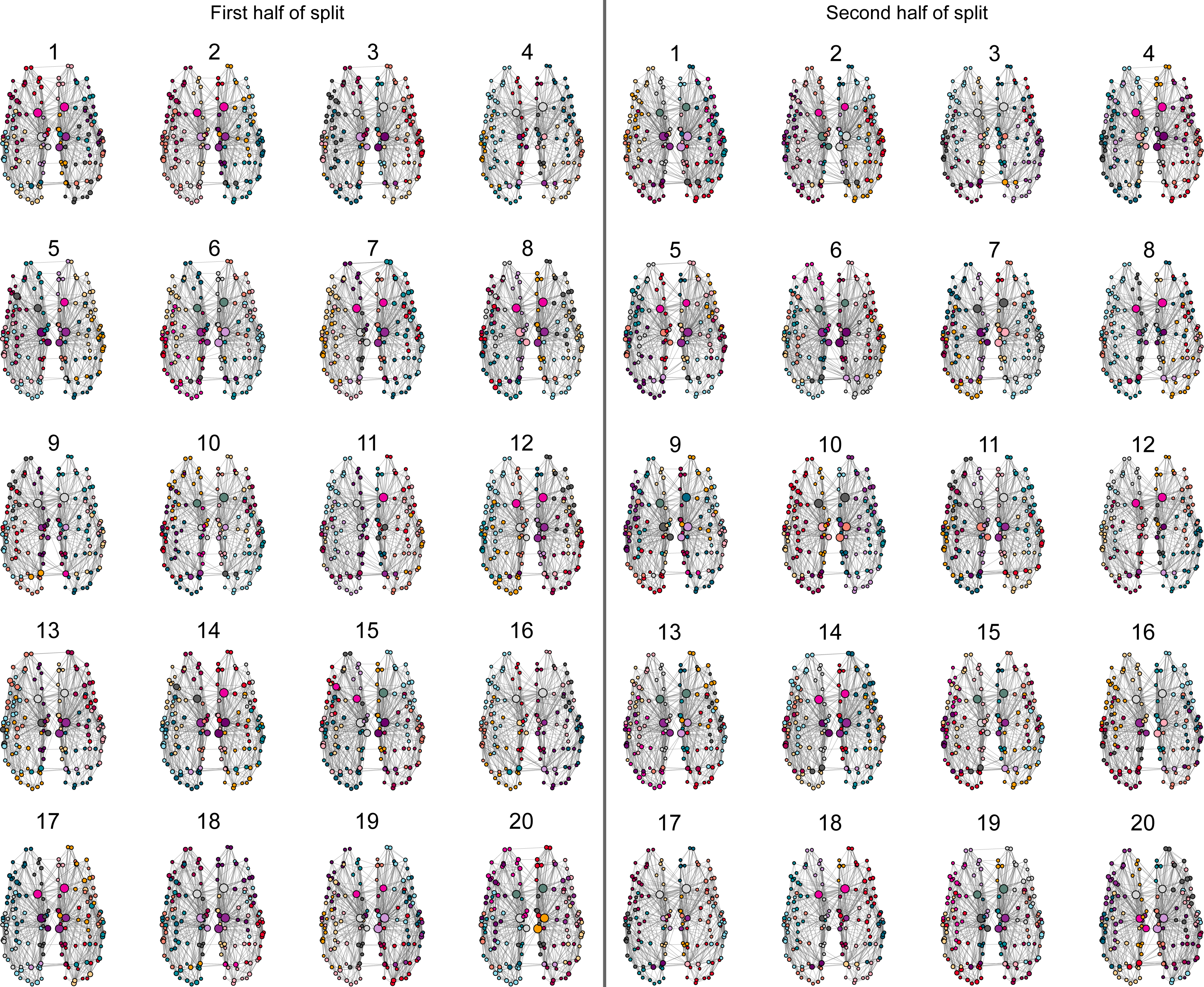

Supplement: S3 Fig — (TIF) [file pone.0117179.s003.tif]

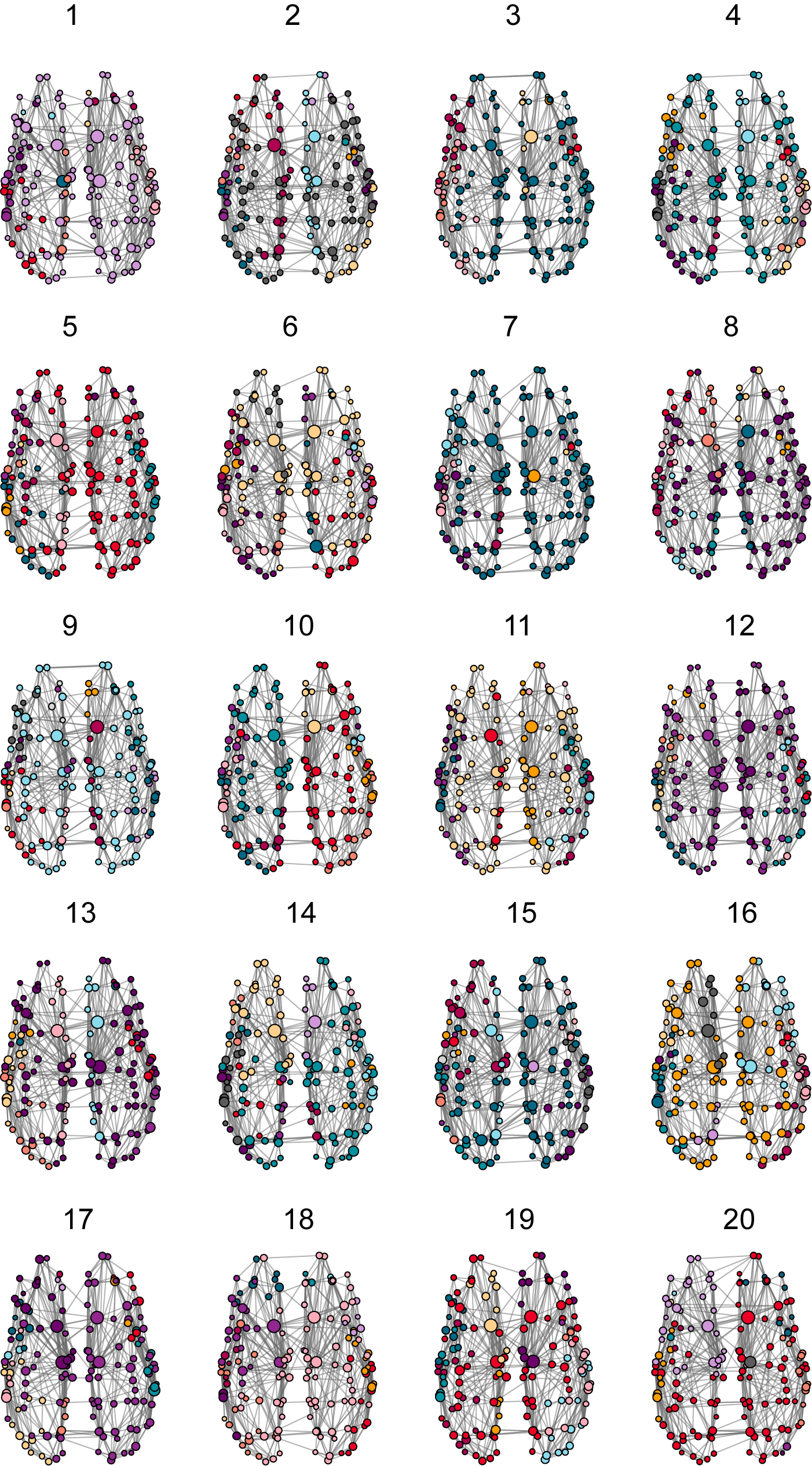

Supplement: S4 Fig — (TIF) [file pone.0117179.s004.tif]

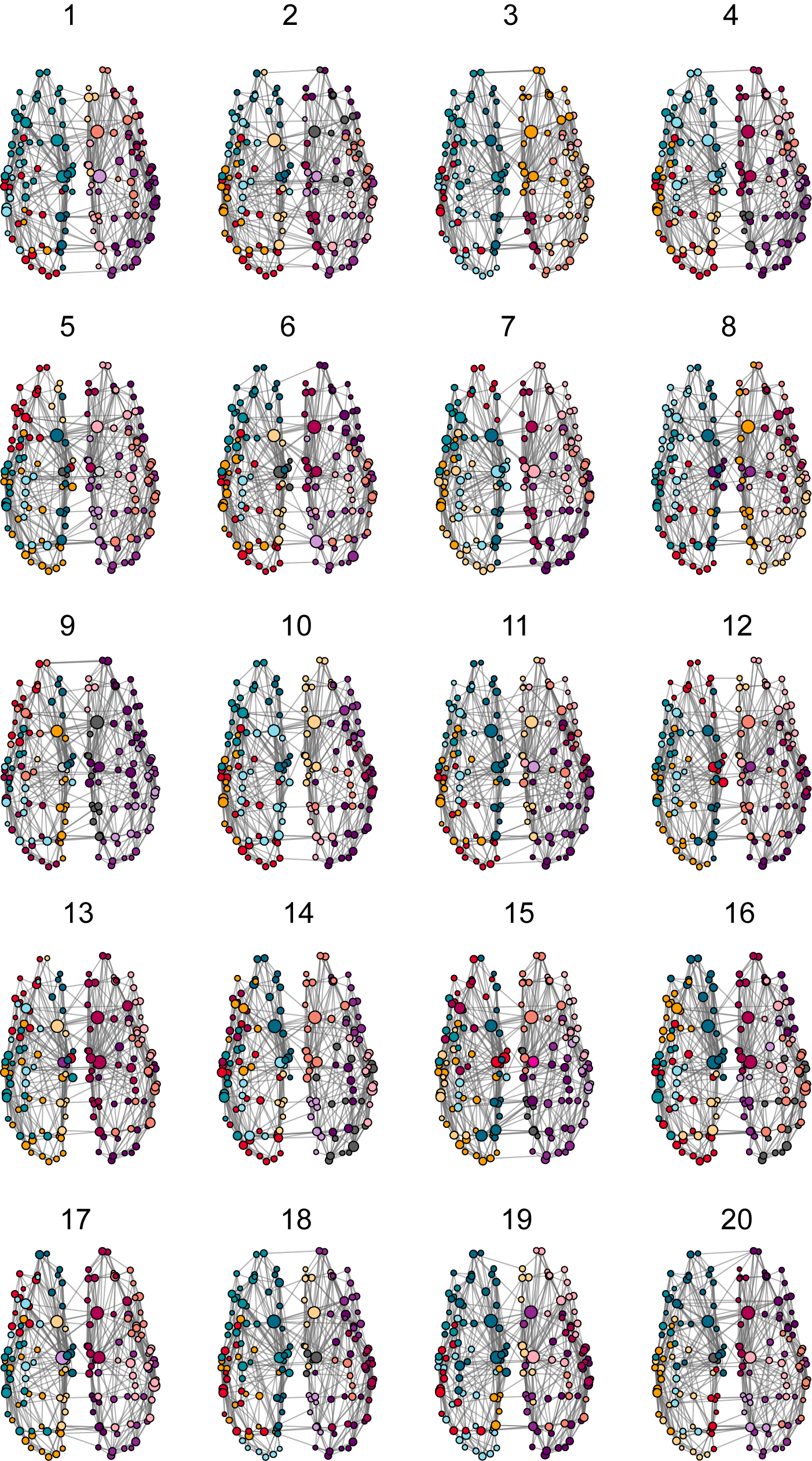

Supplement: S5 Fig — (TIF) [file pone.0117179.s005.tif]

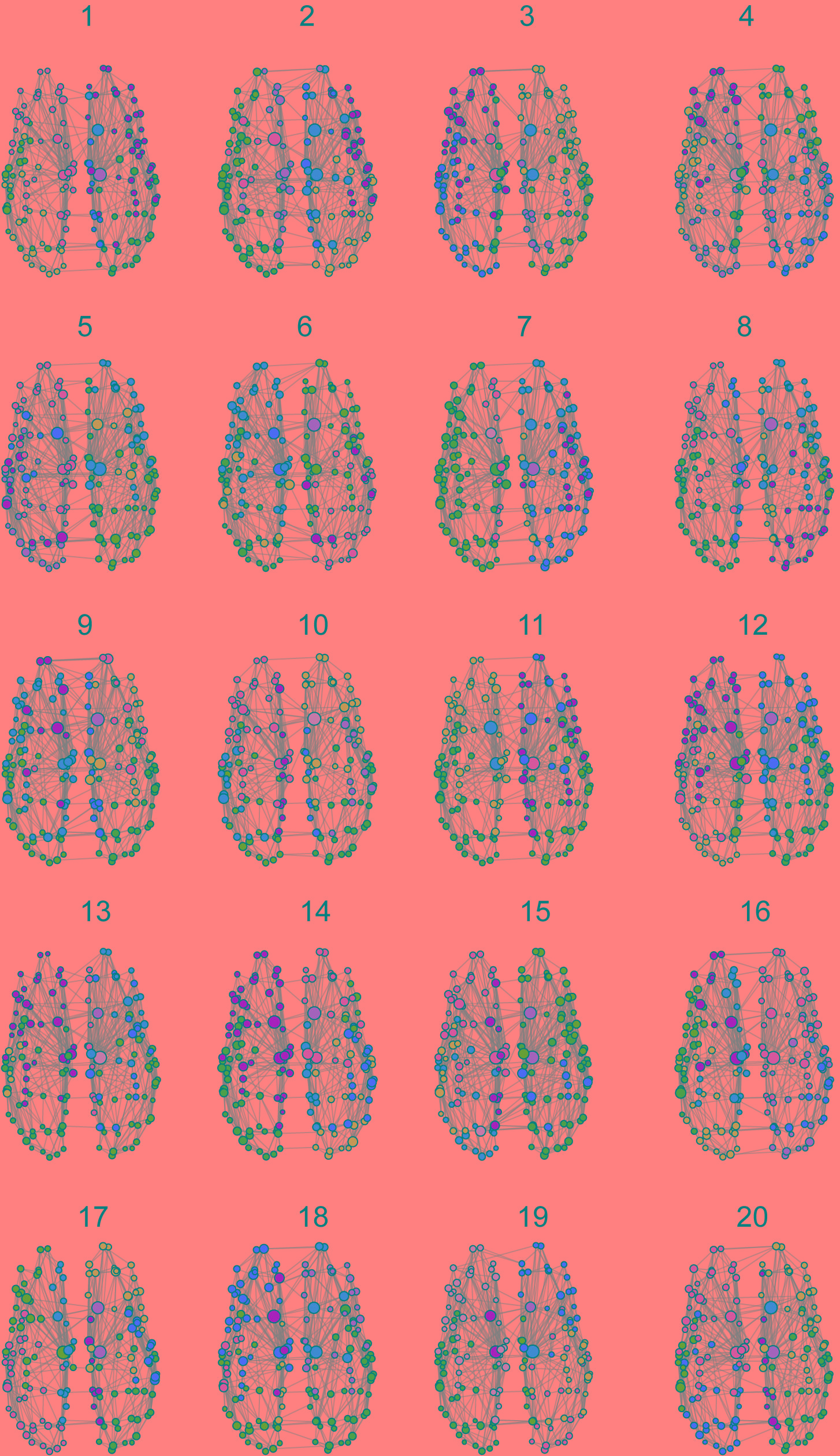

Supplement: S6 Fig — (TIF) [file pone.0117179.s006.tif]
