# Supplementary material for: Probabilistic Clustering of the Human Connectome Identifies Communities and Hubs
Source: PLoS One. 2015 Jan 30;10(1):e0117179. doi: 10.1371/journal.pone.0117179 (PMC4311978; doi:10.1371/journal.pone.0117179)
Supplement: S1 Text — Description of the block Gibbs sampler used to approximate the posterior distributions and maximum a posteriori estimates. (PDF) [file pone.0117179.s007.pdf]

Supporting information 1: inference and parameter selection

1 Inference

In this section we elaborate on the MCMC approximations that we use to infer connectivity $\mathbf{A}$ and clustering $\mathbf{Z}$. In the single-subject case, connectivity and clustering are characterized by the joint posterior distribution

$$P\left(\mathbf{A},\mathbf{Z} \mid \mathbf{S},\delta_T,\delta_F,\alpha,\beta,\xi\right) \propto P\left(\mathbf{S} \mid \mathbf{A},\delta_T,\delta_F\right) P\left(\mathbf{A} \mid \mathbf{Z},\alpha,\beta\right) P\left(\mathbf{Z} \mid \xi\right) \ . \quad (1)$$

First we note that in the definition of $P\left(\mathbf{A} \mid \mathbf{Z},\alpha,\beta\right)$, α and β are used to draw a probability ρ from a beta distribution, which is then used to draw a connection a_{ij} from a Bernoulli distribution. As the beta distribution is conjugate to the Bernoulli, ρ may be integrated out, i.e.

$$\begin{aligned} P\left(\mathbf{A} \mid \mathbf{Z},\alpha,\beta\right) &= \int P\left(\mathbf{A} \mid \boldsymbol{\rho}\right) P\left(\boldsymbol{\rho} \mid \alpha,\beta\right) \mathrm{d}\boldsymbol{\rho} \\ &= \prod_{a\geq b} \frac{\mathrm{Beta}\left(B_+(a,b),B_-(a,b)\right)}{\mathrm{Beta}\left(\boldsymbol{\beta}_+(a,b),\boldsymbol{\beta}_-(a,b)\right)} \end{aligned} \quad (2)$$

with a and b clusters and $B_+(a,b) = M_+(a,b)+\boldsymbol{\beta}_+(a,b)$ and $B_-(a,b) = M_-(a,b)+\boldsymbol{\beta}_+(a,b)$, where $M_+(a,b) = (1-\frac{1}{2}\delta_{ab})\mathbf{z}_a^T\mathbf{A}\mathbf{z}_b$ is the number of edges between regions in cluster a and regions in cluster b, $M_-(a,b) = (1-\frac{1}{2}\delta_{ab})\mathbf{z}_a^T(\mathbf{e}\mathbf{e}^T-\mathbf{I}_n)\mathbf{z}_b - M_+(a,b)$ is the number of non-edges between regions in clusters a and b, $\mathbf{e}$ is a vector of ones of size n, $\mathbf{I}_n$ is the $n\times n$ identity matrix and finally the Beta function defined as $\mathrm{Beta}(a,b) = \int_0^1 x^{a-1}(1-x)^{b-1}\mathrm{d}x,$. The forward model for $\mathbf{S}$ can be simplified in a similar way, as the Dirichlet distribution is the conjugate prior for the multinomial, i.e.

$$\begin{aligned} &P\left(\mathbf{S} \mid \mathbf{A},\delta_T,\delta_F\right) \\ &= \int P\left(\mathbf{S} \mid \mathbf{A},\mathbf{X}\right) P\left(\mathbf{X} \mid \delta_T,\delta_F\right) \mathrm{d}\mathbf{X} \\ &= \prod_i \left[\frac{\left(\sum_j S_{ij}\right)!}{\prod_j S_{ij}!} \frac{\Gamma(\sum_j \delta_{ij})}{\Gamma\left(\sum_j(\delta_{ij}+S_{ij})\right)} \prod_j \frac{\Gamma(\delta_{ij}+S_{ij})}{\Gamma(\delta_{ij})} \right] \ , \end{aligned} \quad (3)$$

with $\delta_{ij} = \delta_T a_{ij} + \delta_F(1-a_{ij})$.

The posterior distribution can be approximated using Gibbs sampling. Conveniently, the different variables can be updated in blocks, which allows us to iteratively update the conditionals

$$\begin{aligned} &P\left(\mathbf{A} \mid \mathbf{Z},\mathbf{S},\delta_T,\delta_F,\alpha,\beta\right) \\ &\propto P\left(\mathbf{S} \mid \mathbf{A},\delta_T,\delta_F\right) P\left(\mathbf{A} \mid \mathbf{Z},\alpha,\beta\right) \\ &= \prod_i \left[\frac{\left(\sum_j S_{ij}\right)!}{\prod_j S_{ij}!} \frac{\Gamma(\sum_j \delta_{ij})}{\Gamma\left(\sum_j(\delta_{ij}+S_{ij})\right)} \prod_j \frac{\Gamma(\delta_{ij}+S_{ij})}{\Gamma(\delta_{ij})} \right] \\ &\quad \times \prod_{a\geq b} \frac{\mathrm{Beta}\left(B_+(a,b),B_-(a,b)\right)}{\mathrm{Beta}\left(\boldsymbol{\beta}_+(a,b),\boldsymbol{\beta}_-(a,b)\right)} \ , \end{aligned} \quad (4)$$

and

$$
\begin{aligned}
&P(\mathbf{Z} \mid \mathbf{A}, \mathbf{S}, \alpha, \beta, \xi) \\
&\propto P(\mathbf{A} \mid \mathbf{Z}, \alpha, \beta)\, P(\mathbf{Z} \mid \xi) \\
&= \left[\prod_{a \geq b} \frac{\mathrm{Beta}\left(B_{+}(a,b), B_{-}(a,b)\right)}{\mathrm{Beta}\left(\boldsymbol{\beta}_{+}(a,b), \boldsymbol{\beta}_{-}(a,b)\right)}\right] \xi^{k} \frac{\Gamma(\xi)}{\Gamma(\xi+n)} \prod_{a} \Gamma(n_a) \ ,
\end{aligned} \tag{5}
$$

in which n_a represents the number of regions in cluster a. The iterative block sampling approach is intuitively straightforward; we first update the connectivity variables given the clustering, then we update the clustering variables given the connectivity. To converge to the desired distribution it is essential that in each iteration the relevant variable is updated according to the most recent estimate of all other variables. We developed a Metropolis sampler for the first conditional [1] and similarly [2] developed a Gibbs sampler for the inference of the IRM, which implements a split-merge sampler to improve mixing [3]. Both samplers are implemented in Matlab.

For the hierarchical setting in which one cluster assignment $\mathbf{Z}$ is inferred for a group of subjects, let again $\boldsymbol{\mathcal{A}} = (\mathbf{A}^{(1)}, \ldots, \mathbf{A}^{(M)})$ and $\boldsymbol{\mathcal{S}} = (\mathbf{S}^{(1)}, \ldots, \mathbf{S}^{(M)})$. The posterior distribution that we are now interested in is given by

$$
\begin{aligned}
&P(\boldsymbol{\mathcal{A}}, \mathbf{Z} \mid \boldsymbol{\mathcal{S}}, \delta_T, \delta_F, \alpha, \beta, \xi) \\
&\qquad \propto P(\boldsymbol{\mathcal{S}} \mid \boldsymbol{\mathcal{A}}, \delta_T, \delta_F)\, P(\boldsymbol{\mathcal{A}} \mid \mathbf{Z}, \alpha, \beta)\, P(\mathbf{Z} \mid \xi) \ .
\end{aligned} \tag{6}
$$

The different subjects are assumed to be conditionally independent. Accordingly, the two conditionals factorize over subjects and become

$$
\begin{aligned}
&P(\boldsymbol{\mathcal{A}} \mid \mathbf{Z}, \boldsymbol{\mathcal{S}}, \delta_T, \delta_F, \alpha, \beta) \\
&\propto P(\boldsymbol{\mathcal{S}} \mid \boldsymbol{\mathcal{A}}, \delta_T, \delta_F)\, P(\boldsymbol{\mathcal{A}} \mid \mathbf{Z}, \alpha, \beta) \\
&= \prod_{m} P\left(\mathbf{S}^{(m)} \mid \mathbf{A}^{(m)}, \delta_T, \delta_F\right) P\left(\mathbf{A}^{(m)} \mid \mathbf{Z}, \alpha, \beta\right) \\
&= \prod_{m} \prod_{i} \left[\frac{\left(\sum_j S_{ij}^{(m)}\right)!}{\prod_j S_{ij}^{(m)}!} \frac{\Gamma(\sum_j \delta_{ij}^{(m)})}{\Gamma\left(\sum_j (\delta_{ij}^{(m)} + S_{ij}^{(m)})\right)} \right. \\
&\quad \left. \times \prod_{j} \frac{\Gamma(\delta_{ij}^{(m)} + S_{ij}^{(m)})}{\Gamma(\delta_{ij}^{(m)})} \right] \\
&\quad \times \prod_{a \geq b} \frac{\mathrm{Beta}\left(B_{+}^{(m)}(a,b), B_{-}^{(m)}(a,b)\right)}{\mathrm{Beta}\left(\boldsymbol{\beta}_{+}(a,b), \boldsymbol{\beta}_{-}(a,b)\right)} \ ,
\end{aligned} \tag{7}
$$

with $\delta_{ij}^{(m)} = \delta_T a_{ij}^{(m)} + \delta_F (1 - a_{ij}^{(m)})$, and

$$
\begin{aligned}
&P(\mathbf{Z} \mid \boldsymbol{\mathcal{A}}, \boldsymbol{\mathcal{S}}, \alpha, \beta, \xi) \\
&\propto P(\boldsymbol{\mathcal{A}} \mid \mathbf{Z}, \alpha, \beta)\, P(\mathbf{Z} \mid \xi) \\
&= \left[\prod_{m} P\left(\mathbf{A}^{(m)} \mid \mathbf{Z}, \alpha, \beta\right)\right] P(\mathbf{Z} \mid \xi) \\
&= \left[\prod_{m} \prod_{a \geq b} \frac{\mathrm{Beta}\left(B_{+}^{(m)}(a,b), B_{-}^{(m)}(a,b)\right)}{\mathrm{Beta}\left(\boldsymbol{\beta}_{+}(a,b), \boldsymbol{\beta}_{-}(a,b)\right)}\right] \\
&\quad \times \xi^{k} \frac{\Gamma(\xi)}{\Gamma(\xi+n)} \prod_{a} \Gamma(n_a) \ .
\end{aligned} \tag{8}
$$

To sample from the joint distribution in the hierarchical case, one simply iterates over updating the connectivity for each subject given the group-clustering and updating of clusterlation given the product of the connectivity for each subject.

When approximating these posterior distributions using MCMC, one needs to assess whether the sampling chains have converged to the true distribution. We analyzed convergence by calculating the potential scale reduction factor (PSRF) [4], which can be understood as a ratio between intra-chain variance and inter-chain variance. Heuristically, we assumed convergence for connectivity when for each edge a_{ij} the PSRF dropped below 1.1. Convergence for clustering was expressed by deriving for each sample of $\mathbf{Z}$ the cluster probability matrix $\mathbf{M} = \mathbf{Z}^T\mathbf{Z}$. Similar to the convergence of connectivity, we assumed that the samples were converged to the target distribution when the PSRF for each m_{ij} was smaller than 1.1. To be able to calculate the PSRF, 10 independent sampling chains were executed, each starting from different, randomized initializations, consisting of a draw from the Chinese restaurant process for $\mathbf{Z}$ and an Erdős-Rényi random graph for $\mathbf{A}$ with a density of 20%. We found that 2,000 Gibbs iterations were sufficient to reach convergence. Note that for each iteration every element a_{ij} and z_{ir} was updated once. For the experiments that involved maximum a posteriori estimates instead of probability distributions, both the samplers were altered to perform simulated annealing with an exponential decay cooling schedule, which enforces convergence towards the mode of the distribution.

The computational complexity of one iteration of the bIRM sampler is $O(mMK^2)$ with $m \leq N^2$ the number of edges. Sampling connectivity for one subject for one iteration has a time complexity of $O(N^2)$. The sIRM approach therefore scales as $O(MN^2K^2)$, or, if we assume $K \ll N$, as is the case in the parcellations we find, $O(MN^2)$ instead. For comparison, the Infomap algorithm has a time complexity of $O(m)$ and K-means runs in $O(N^2K)$. This makes the proposed approach the slowest of the three algorithms. Note however that it provides the posterior distributions of $\mathbf{A}$, $\mathbf{Z}$ and $\boldsymbol{\rho}$ instead of a point estimate of $\mathbf{Z}$, and that the sampling process includes the determination of (the distribution of) K.

References

[1] M. Hinne, T. Heskes, C. F. Beckman, and M. van Gerven. Bayesian inference of structural brain networks. *NeuroImage*, 66:543–552, 2013.

[2] M. Mørup, K. H. Madsen, A.-M. Dogonowski, H. Siebner, and L. K. Hansen. Infinite relational modeling of functional connectivity in resting state fMRI. In *Neural Information Processing Systems*, volume 23 of *NIPS'10*, pages 1750–1758. Curran Associates, Inc., 2010.

[3] S. Jain and R. M. Neal. Splitting and merging components of a nonconjugate Dirichlet process mixture model. *Bayesian Anal.*, 2(3):445–472, 2007.

[4] Andrew Gelman and Donald B. Rubin. Inference from iterative simulation using multiple sequences. *Stat Sci*, 7(4):457–472, 1992.
