# Supplementary material for: Probabilistic Clustering of the Human Connectome Identifies Communities and Hubs
Source: PLoS One. 2015 Jan 30;10(1):e0117179. doi: 10.1371/journal.pone.0117179 (PMC4311978; doi:10.1371/journal.pone.0117179)
Supplement: S2 Text — (PDF) [file pone.0117179.s008.pdf]

Supporting information 2: parameter selection

1 Parameter selection

A number of parameters potentially influence the parcellations we obtain. These are

1. ξ, the concentration parameter of the Chinese restaurant prior,
2. α and β that determine the expected probability of a connection between two clusters via a beta distribution and
3. the two different Dirichlet hyperparameters δ_T and δ_F that influence how many streamlines are observed due to noise.

Throughout our experiments we used $\xi = \log N$, cf. [1]. We observed that our results where very robust against different settings of ξ. In fact, only when ξ was increased to result in an expected number of clusters that was orders of magnitudes larger than the number of nodes (which makes it an impossible parcellation), did this parameter seem to have an effect. For the parameters α and β, the choice of $\alpha = \beta = 1$ was determined by our experimental setup, in which we did not want to make any prior assumptions on the cluster connectivity behavior in our parcellations. Finally, the parameters δ_T and δ_F determine the relation between observed streamlines and structural connectivity. The chosen setting of $\delta_T = 1$ and $\delta_F = 0.1$ results in an uninformative prior for connected pairs of nodes, while enforcing that high streamline counts are unlikely to be explained by non-connections. This intuition was previously validated, by comparing the estimates for structural connectivity with functional independencies [2].

References

[1] M. Mørup, K. H. Madsen, A.-M. Dogonowski, H. Siebner, and L. K. Hansen. Infinite relational modeling of functional connectivity in resting state fMRI. In *Neural Information Processing Systems*, volume 23 of *NIPS'10*, pages 1750–1758. Curran Associates, Inc., 2010.

[2] M. Hinne, T. Heskes, C. F. Beckman, and M. van Gerven. Bayesian inference of structural brain networks. *NeuroImage*, 66:543–552, 2013.
