# Supplementary material for: Probabilistic Clustering of the Human Connectome Identifies Communities and Hubs
Source: PLoS One. 2015 Jan 30;10(1):e0117179. doi: 10.1371/journal.pone.0117179 (PMC4311978; doi:10.1371/journal.pone.0117179)
Supplement: S3 Text — (PDF) [file pone.0117179.s009.pdf]

Supporting information 3: K-means and Infomap

1 K-means and Infomap

The K-means implementation that comes with the Matlab (MATLAB 7.7, The MathWorks Inc., Natick, MA, USA) Statistics Toolbox was used with the cosine similarity as the (inverse) distance metric. The Infomap algorithm [1] was implemented using code from the Graph Cluster toolkit [2]. Both procedures were repeated 300 times with random initializations, of which the best was used as the final result.

References

[1] M. Rosvall and C. T. Bergstrom. Maps of random walks on complex networks reveal community structure. *Proc Natl Acad Sci USA*, 105(4):1118–1123, 2008.

[2] T. van Laarhoven and E. Marchiori. Graph clustering with local search optimization: The resolution bias of the objective function matters most. *Phys. Rev. E*, 87:012812, 2013.
